# Ellagic acid attenuates post-cerebral ischemia and reperfusion behavioral deficits by decreasing brain tissue inflammation in rats

**DOI:** 10.22038/ijbms.2020.41821.9882

**Published:** 2020-05

**Authors:** Khadijeh Hassonizadeh Falahieh, Alireza Sarkaki, Mohammadamin Edalatmanesh, Mohammad Kazem Gharib Naseri, Yaghoob Farbood

**Affiliations:** 1 Department of Physiology, College of Sciences, Science and Research Branch, Islamic Azad University, Fars, Iran; 2 Department of Physiology, College of Sciences, Shiraz Branch, Islamic Azad University, Shiraz, Iran; 3 Persian Gulf Physiology Research Center and Department of Physiology, Ahvaz Jundishapur University of Medical Science, Ahvaz, Iran; 4 Medicinal Plants Research Center, Ahvaz Jundishapur University of Medical Science, Ahvaz, Iran

**Keywords:** Anxiety-like behavior, BBB permeability, Brain edema, Cerebral, ischemia/reperfusion, Cytokines, Depression-like behavior, Ellagic acid, Exploratory behaviors

## Abstract

**Objective(s):**

Cerebral ischemia/reperfusion (I/R) causes brain inflammation that ultimately causes long time brain function disturbances. We aimed to evaluate the effect of ellagic acid (EA) on anxiety, depression, locomotion behaviors, blood-brain barrier (BBB) permeability, brain edema, and inflammation in male rats with cerebral I/R.

**Materials and Methods:**

Sixty male Wistar rats (250-300 g) divided into 6 groups randomly with 10 in each: 1) Sham+Veh; rats submitted to the surgery without any I/R and received vehicle (10% DMSO in normal saline 5 ml/kg, gavages). 2) I/R+Veh; 3-5) I/R+EA; I/R rats received 50, 75 and 100 EA mg/kg, by gavages 3 times daily for one week. The cerebral I/R injury was induced by clamping the bilateral common carotid arteries for 20 minutes followed by reperfusion. Behaviors were tested one week after treatment, and brain tissue cytokines were measured by special ELISA kits.

**Results:**

Cerebral I/R disrupted BBB function (*P*<0.001), increased brain water content (*P*<0.01), anxiety-like (*P*<0.001), depression-like (*P*<0.001) behaviors and cytokines in the brain tissue (*P*<0.001), while decreased locomotion and exploratory behaviors significantly (*P*<0.01 and *P*<0.001, respectively). Administration of EA (100 mg/kg but not other doses) could improve post-ischemic complications such as clinical signs (*P*<0.01), BBB function (*P*<0.001), brain edema (*P*<0.01), brain tissue cytokines (*P*<0.001), locomotion and exploratory behaviors significantly (*P*<0.05 and *P*<0.001, respectively).

**Conclusion:**

The results suggest that EA could be a potential therapeutic agent against cerebral I/R, possibly through its intertwined anti-inflammatory effects. Further research is required to investigate the involved mechanisms in details.

## Introduction

Stroke is the third leading cause of death after cardiovascular disease and cancer in the world (1). Eighty-five percent of stroke patients are ischemic stroke caused by occlusion or narrowing the brain blood vessel (2). In ischemic stroke, a significant reduction in local cerebral blood flow leads to deprivation of oxygen and glucose, leading to brain damage. During reperfusion, increasing the amount of oxygen is crucial for the existence of the nerve cells. However, the pro-oxidant enzyme and mitochondria also use oxygen as a substrate to produce significant amounts of free radicals (oxidants) during reperfusion (3). Brain tissue has high sensitivity to ischemic injury due to its high metabolism and low oxygen reserves (4). The brain accounts for only 2% of the total body weight (about 1.3 to 1.4 kg), while consumes about 20% of the oxygen up-take by body thereby produces more free radicals than other organs. In addition, brain tissue contains significant amounts of fats with unsaturated fatty acids and high iron concentrations, so the brain is more vulnerable to free radical damage (5). Reducing the oxygen concentration of the cell leads to decrease in adenosine triphosphate (ATP). In this case, the cell uses anaerobic respiration to produce biological energy to survive, which causes accumulation of lactate and then acidosis and cell death (6, 7). Combining these radicals with nitric oxide radicals produces a very dangerous compound in the ischemic region called peroxynitrite with an important role in damage to the nerve tissue, including the formation of tissue necrosis and excitation of cellular death (8). Basic and clinical studies demonstrate that the production of free radicals is one of the most important factors that increase inflammatory and oxidative factors following the brain stroke (9). Following the reduction or stop of the cerebral blood flow (CBF), the energy required to maintain the active transportation of sodium and potassium (Na^+^/ K^+^ ATPase) is inhibited due to decreased production of ATP causing imbalance in multiple ions, including Na^+^, Cl^-^, and Ca^2+ ^(10). Several types of cells, including endothelial cells, astrocytes, microglia and neurons help to induce the post-ischemic inflammation (11). The pathophysiology of stroke involves stimulation of the excitotoxicity mechanisms, neuronal inflammatory and oxidative pathways and ionic imbalance in the brain cells. Different mechanisms that interfere with brain tissue damage occur in two stages of ischemia caused by a decrease in the blood flow in the respective position, and in the next stage, which is called reperfusion, that is caused by the return of the higher blood flow to the tissue after a period of ischemia (12). As a result, disturbances are created at the level of the structure and function of the cell components. These include disorders of mitochondrial function, disturbances in the activity of ionic pumps in plasma membranes, and structural and functional destructions in the brain tissue. The total of these damages is called waste of reperfusion or I/R (13). These injuries are mainly caused by two mechanisms of increased inflammatory reactions and oxidative stress. In fact, in the brain tissue after an I/R, a cascade of vascular permeability, an increase in cellular signaling and an increase in the penetration of circulating leukocytes occur in damaged tissue. Approximately 3 min after I/R, leukocytes rapidly attack to the ischemic site that causes inflammatory response. This inflammatory response has been observed both in empirical models and in human specimens that primarily interfere with the destruction of tissue and organs (14, 15). 

Therefore, the use of natural substances or herbal medicines with potent anti-oxidative and anti-inflammatory properties to improve the neurodegenerative complications such as traumatic brain injury (TBI), cerebral I/R, Parkinson’s and Alzheimer’s diseases has been suggested. They may be able to somehow protect the brain against damages from oxidants and inflammatory agents following the stroke to reduce neuronal mortality (16-18).

Ellagic acid (EA) is a dimeric derivative of gallic acid, which is found mainly in plants (19, 20). The highest level of EA is found in the raspberry, and also in many red fruits such as strawberries, pomegranates, blackberries, persimmons, grapes, peaches, plums, walnuts and pecans nuts (20, 21). EA showed many useful effects, including anti-oxidative, anti-inflammatory and anti-tumor regulation and anti-proliferative effects in rats with TBI, but its mechanisms at cellular and molecular level is still unknown (17, 22). EA has a potential to induce apoptosis or cell death in cancer cells (23). 

Regarding to available knowledge about the complications following the brain I/R and the beneficial effects of natural substances derived from plants, in current study we aimed to investigate the therapeutic effects of EA on the brain oxidative stress and inflammation due to blood-brain barrier (BBB) disruption and brain edema followed by behavioral deficits in an experimental model of I/R in rats. So, in this study the anxiety, depression and motor activity behaviors*,* and brain tissue inflammation in male rats with cerebral I/R were investigated.

## Materials and Methods

***Animals***

Sixty male Wistar rats weighing 250- 300 g were purchased from main animal care and breeding center of Ahvaz Jundishapur University of Medical Sciences (Ahvaz, Iran) and divided randomly in 6 groups with 10 in each. Each rat housed in standard individual cage, under room temperature of 20 to 22 ^°^C, 12 hr dark/light cycle (lighting at 7 AM), 50% to 55% humidity and free access to food (chow pellets) and top water. All experiments were performed in accordance with Ethics guides approved by Ethics Committee of Islamic Azad University of Shiraz, Fars (IR-iaushiraz-95-11-758).

Animals divided randomly into 6 groups; 1) Sham+Veh group, which consumed ordinary food and submitted to the surgery without the occlusion of bilaterally common carotid arteries, received 5 ml/kg DMSO/ normal saline (10%) as solvent of EA, three times daily in the morning, afternoon and night at intervals of eight hours for one week.

2) I/R+Veh group; I/R rats received 5 ml/kg DMSO/normal saline (10%), three times daily for one week.

**Figure 1:**
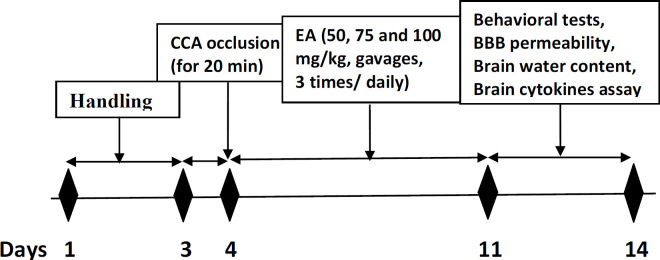
Schematic diagram of experimental protocol. CCA; common carotid artery, BBB; blood brain barrier

3-5) I/R+EA groups; I/R rats received 50, 75 or 100 mg/kg EA respectively, three times daily for one week (16, 17). 6) Positive Cont (+Cont); intact rats received the best effective dose (100 mg/kg, gavages). In this group, we aimed to determine what is the effect of EA (best effective dose) on healthy animals to determine whether has additive or decreasing effects on studied parameters? Time line and experimental design of all protocols are shown in Figure 1. 

***Induction of cerebral ischemia/reperfusion (I/R)***

Rats were anesthetized with ketamine/xylazine (90:5 mg/kg, IP). A neck ventral midline incision was made, and the right and left carotid arteries were then exposed and gently separated from the vagus nerves. They were occluded for 20 minutes using the mini vessel clamps. After that, the clamps were removed and the blood flowed back to the brain**. **This surgery causes a reversible obstruction of common carotid arteries. Mortality rate due to I/R surgery was about 8-10 percents. After this stage, the slit was sutured and the animals were placed in their individual cages. After successful induction of cerebral I/R, some related neurological signs such as loss of consciousness and righting reflex were evaluated to prove its complications (24, 25)**.**

***Evaluation of neurological signs***

Neurological outcomes were assessed according to methods of Garcia (26). For this purpose, 72 hr after I/R, each animal in all tested groups was examined for six behavioral parameters.

*Spontaneous activity*

Each animal is observed for 5 min, while it is in the cage. The movement of rats was assessed by their ability to approach all four walls of the cage and scored as the following: 0: the rats did not have activity at all; 1: the rats that severely affected barely moved in the cage, and did not rise up; 2: slightly affected rats slowly moved in the cage, but did not approach all sides and reached at least one upper rim of the cage; and 3: rats explored the environment, moved around, and approached at least three walls of the cage.

*Symmetry in the movement of four limbs*

Each rat was held in the air by the tail to evaluate symmetry in the four limbs movements, and scored as follows: 0: forelimb on the left side did not move at all; 1: minimal movements observed in the left side limbs; 2: the left side limbs extended less or more slowly than the right limbs; 3: all four limbs extended symmetrically.

*Forepaw outstretching*

Each rat was brought up to the edge of the table, while being held by the tail and allowed to walk on forelimbs and scored as follows: 0: the left forelimb did not move; 1: the left forelimb moved minimally; 2: the right side outstretched more than the left, and forepaw walking was impaired; and 3, the rat walked symmetrically on forepaws, while both forelimbs were outstretched.

*Climbing*

Rats were placed on the wall of a wire cage and scored as follows: 1: the rat tended to circle instead of climbing and failed to climb; 2: the left side did not grip as hard as the right side, or it was impaired while climbing; 3: the rat gripped tightly to the wire and climbed easily.

*Body proprioception*

Each side of the rat’s body was touched with a blunt stick, and the reaction to the stimulus was observed and scored as the following: 1: the rat did not react to left-sided stimulation; 2: the rat reacted slowly to left-sided stimulation; and 3: the rat responded to the stimulus by turning head, and it was equal on both sides.

*Response to touching vibrissae*

A blunt stick was brushed against the vibrissae on each side of the body; to avoid entering the visual fields, the stick was moved toward the whiskers from the rear of the rat. The scoring was as follows: 1: the rat did not react to left-sided stimulation; 2: the rat responded slowly to left-sided stimulation; and 3: the rat responded to the stimulus by head turning, and it was equal on both sides.

***Evaluation of anxiety–like behavior***

The black metal elevated plus maze device (Borj Sanat Co, Tehran, Iran) was used to measure the anxiety level in the rats. This device consists of two open arms with dimensions of 0.25 × 10 × 50 cm and two closed arms with dimensions of 15 × 10 × 50 cm and a central portion of 10 × 10 cm. While the animal’s face was directed toward the open arm, rats placed in the central square. The animal allowed to explore in the device for 5 minutes. The animal’s path was recorded by the software (Ethovision software, version 7, Noldus Co. Netherland). Tested parameters including the number of entries and time spent into the open arms were recorded. To prevent the effect of smell due to the presence of rat in the previous experiment, the floor and the walls of the EPM were cleaned with 10% alcohol solution (27). 

***Locomotion activity test***

This test was used to assess behavioral responses such as motor activity, hyperactivity and exploratory behaviors. The open field device (Borj Sanat Co, Tehran, Iran) consisted of a galvanized sheet box (60 * 20 * 60) with black colored floor and walls. The floor of the device was divided into16 equal squares by software (Ethovision software, version 7, Noldus Co. Netherland). Each rat at the first day was individually placed in the center of the arena, and allowed to freely explore inside the maze. The next day, it was placed in the cleaned arena again for 5 min. During this time, the animal’s activities were recorded via video-tracking system. Ambulation (crossed lines) and, exploratory behavior including standing upon the rear limbs, and touching the surrounding walls with claws (rearing to explorer outside of maze), the time spent in the central square and freezing time in arena were recorded. The device was carefully cleaned with 10% ethanol solution after each test (28). 

***Depression-like behavior evaluation***

The forced swimming test in a Plexiglas cylinder (60 cm height, 25 cm inner diameter (Omid-Iranian Co, Tehran, Iran)), filled up to 30 cm high of water (25 ^°^C ) was use to evaluate the depression–like behavior. Each animal was placed individually inside the forced swimming cylinder for 5 min as fast swimming test (FST), and immobile and floating behaviors were recorded during this time. Immobilization involves the inactive behavior of the animal in cylinder, which is defined only to keep the head out of the water and the swimming included active behavior in water other than holding the head of water (for example, swimming around the cylinder) (27). 

***Measuring the brain Evans blue content as a BBB permeability index***

Assessment of BBB permeability was performed by Evans blue (EB) extravasation technique (29). One week after surgery in the sham and ischemic groups, rats received Evans blue solution (2 ml/kg 2%, at the 37 ^°^C) via intravenous slowly injection in tail for 5 min. Twenty four hours after treatment of I/R, animals were deeply anesthetized and kept in a warm environment, then 300 ml warm saline (37 ^°^C) perfused transcardially with blood returning to the right atrium was colorless and transparent. Their brains removed from the skull and one hour later the brain tissue was homogenized in phosphate buffer saline (PBS) adding trichloroacetic acid and was then mixed thoroughly by vortex. The mixture centrifuged for 30 min (3500 rpm) and absorbance of supernatant was evaluated by spectrophotometer at wavelength of blue light (620 nm). Standard curve was used to calculate the concentrations of Evans blue as microgram per gram tissue (μg/g tissue). 

***Percentage of brain water content***

Rats in different groups were anesthetized and decapitated. Their brain carefully removed from the skull. Dry weight / wet weight method was used to measure cerebral edema that was previously described in detail by Gerriets *et al* (30). Brain tissue was placed in container (which had been previously weighed by digital scale) and wet weight (WW) was measured. The brain was placed in the oven for 24 hr at 110 ^°^C to dry. After this period, the dry weight was measured by digital scale. The water content percentage (%Brain Water Content; BWC %) was calculated from the following formula. 

BWC% = [(WW-DW)/WW] x 100

***Measurement of cytokines levels in brain tissue***

After cerebral ischemia and removing the brains according to mentioned above procedure, on the test day, the samples were homogenized in a PBS solution proportional 1/10 and centrifuged at -4 ^°^C and 3000 g for 15 min. Then, supernatant was used to measure the cytokines (31). The brain tissue contents of interleukin- 1 beta (IL-1β) and tumor necrosis factor alpha (TNF-α) were measured using the specific ELISA kits (ZellBio Gmbh, Germany). As briefly, the specimens were poured into wells that contained anti IL-1β antibody. Then, the conjugated secondary antibody was added to the medium with biotin. After adding streptavidin- horseradish peroxidase, it was incubated at 37 ^°^C for 60 min. Then, it was rinsed 5 times by saline. The chromogen was added, and 30 minutes later the stopping solution was added to the medium. The changed color of chromogen was read at the 450 nm optical range. Brain content of IL-1β and TNF- α were expressed in picograms in 1 mg of protein (32). 

***Statistics analysis***

All data expressed as Mean±SEM and were analyzed by SPSS-ver.20 software using one-way ANOVA followed by Tukey’s *post hoc* test. Differences between groups with *P* less than 0.5 assigned as significant. 

## Results

In this work, the effects of EA (50, 75 and 100 mg/kg) on all BBB function, brain edema and brain inflammatory cytokines parameters were assessed. The 100 mg/kg EA had the best effect on above parameters. On the other hand, we administered the best effective dose of EA to 10 intact rats as a positive control group, but there were not any difference with sham group. So, we represented the effects of all used doses of EA on BBB permeability, brain water content (n=5 in each group) and cytokines. 

***Neurological signs scores***

**Figure 2:**
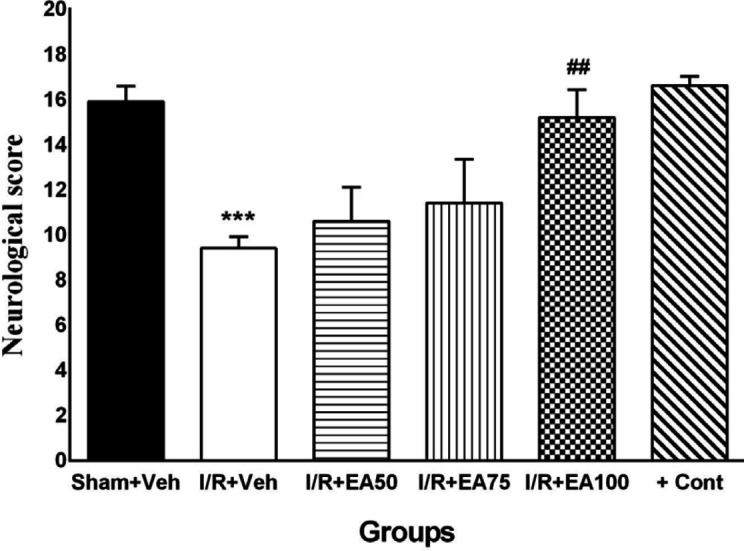
Neurological scores at 72 hr after ischemia/reperfusion. Data were presented as Mean±SEM. (n=10). I/R; ischemia and reperfusion, Veh; vehicle, EA: Ellagic acid, cont; control. ***P<0.001 vs. sham+Veh group. ##*P*< 0.01 vs. I/R+Veh group

The scores obtained from the neurological tests in all experimental groups were shown in Figure 2. Neurological scores were significantly decreased in the I/R+Veh group compared to Sham+Veh (*P*<0.001). Doses 50 and 75 of EA could not change clinical signs, while EA with dose100 increased the signs significantly in comparison with I/R+Veh group (*P*<0.01). 

***Depression-like behavior with FST***

**Figure 3:**
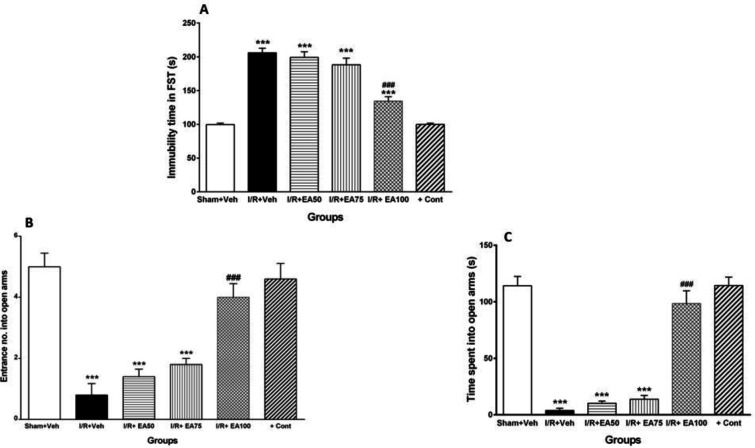
Evaluation of the immobilization time (A) and anxiety- like behavior (B-C) of the animals in the forced swimming and elevated plus maze tests, respectively. Data were represented as Mean±SEM (n=10). I/R; ischemia and reperfusion, Veh; vehicle, EA: Ellagic acid, cont; control. ***P <0.001 vs. sham+Veh group. ###*P*<0.001 vs. I/R+Veh group

Figure 3A shows comparison of the immobilization time of the animals in the forced swimming test. It was significantly increased in I/R+Veh group compared to Sham+Veh (*P*<0.001). Administration of EA (higher dose, 100 mg/kg) decreased it significantly in comparison with I/R+Veh group (*P*<0.001).

***Anxiety-like behavior***

Figure 3 (B-C) illustrates the entrance number (B) and time spent (s) (C) of the tested groups into the open arms of elevated plus maze as anxiety-like behavior measurement. Entrance number and time spent were significantly decreased in I/R+Veh group compared to the sham+Veh (*P*<0.001). Administration of EA (100 mg/kg) restored them significantly when compared to I/R+Veh (*P*<0.001).

***Locomotion and exploratory behaviors***

**Figure 4:**
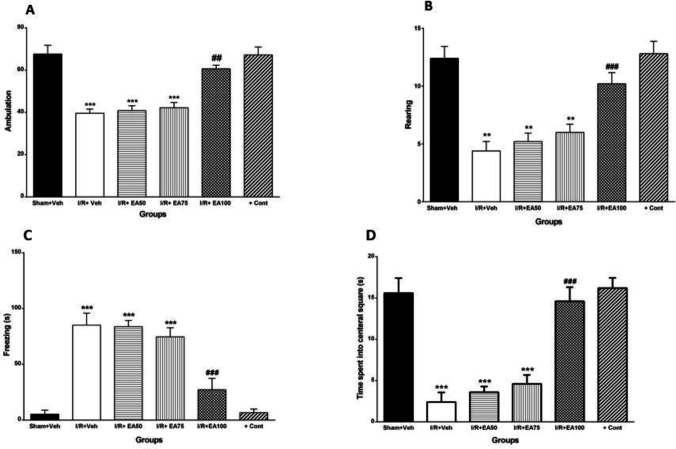
Evaluation of the ambulation or line crossing as locomotion behaviors with low-anxiety (A), rearing as exploratory behavior (B), freezing as locomotion less in a corner square (C) and time spent in central square as daring behavior with no fear and panic (D) of the animals in the open field test. Data were represented as Mean±SEM (n=10). I/R; ischemia and reperfusion, Veh; vehicle, EA: Ellagic acid, cont; control. **P<0.01 and *** P<0.001 vs. Sham+Veh. ##* P*<0.01 and ### *P*<0.001 vs I/R+Veh group

Figures 4 (A-D) shows locomotion and exploratory behaviors in open field test in all tested groups. The ambulation or crossed lines (Figure 4A) and rearing of the animals in arena (Figure 4B) decreased significantly (*P*<0.01). Freezing time (Figure 4C) was increased (*P*<0.001), while time spent in central square (Figure 4D) significantly decreased in I/R+Veh group compared to Sham+Veh group (*P*<0.001). Administration of EA (100 mg/kg) reversed all those tested behaviors significantly versus I/R+Veh (*P*<0.01 for ambulation, and *P*<0.001 for three other behaviors).

***BBB permeability***

**Figure 5.**
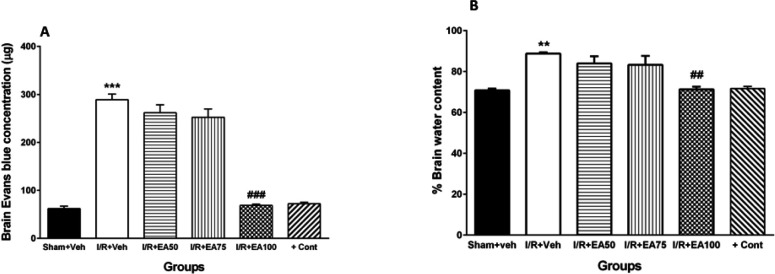
Evans blue extravasations (A) and brain water content (B) of the experimental groups. Data were represented as Mean±SEM (n=5). I/R; ischemia and reperfusion, Veh; vehicle, EA: Ellagic acid, cont; control. **P<0.01 and *** *P*<0.001 vs. Sham+Veh. ## *P*<0.01 and ### *P*<0.001 vs. I/R+Veh group

Figure 5A shows comparison of Evans blue concentration in brain tissue (as BBB permeability index) in all tested groups. It was significantly increased in I/R+Veh group compared to the Sham+Veh (*P*<0.001). Administration of lower doses of EA (50 and 75) could not affect it while the 100 mg/kg EA decreased it significantly when compared to I/R+Veh (*P*<0.001).

***Brain water content***

Figure 5B demonstrates the percent of water content of brain tissue (as brain edema index) in all tested groups. It was significantly increased in I/R+Veh group versus Sham+Veh group (*P*<0.01). Administration of lower doses of EA had no significant effects, while the 100 mg/kg decreased it significantly when compared to I/R+Veh group (*P*<0.01). 

***Brain tissue TNF-α level***

**Figure 6:**
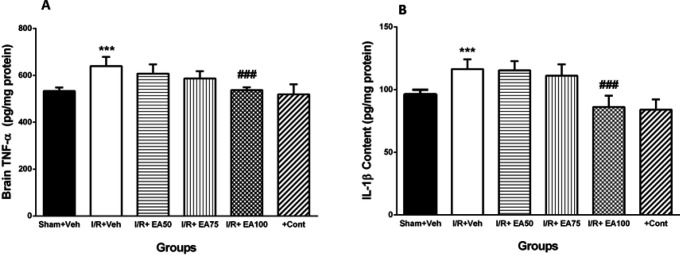
The brain tissue levels of TNF-α (A) and IL-1β (B) following ischemia and ischemia/reperfusion.. Data were represented as Mean±SEM (n=10). IL-1β; Interleukin- 1 beta, TNF-α; Tumor necrosis factor alpha, I/R; ischemia and reperfusion, Veh; vehicle, EA: Ellagic acid, cont; control. *** *P*<0.001 vs. Sham+Veh, ### *P*<0.001 vs. I/R+Veh group

Figure 6A illustrates comparison of the brain content of TNF-α in different tested groups. It was significantly increased in I/R+Veh group compared to the sham+Veh group (*P*<0.001). Administration of lower doses of EA had no significant effects, while the 100 mg/kg EA (I/R+EA100) decreased it significantly related to I/R+Veh group (*P*<0.001).

***Brain tissue IL-1β level***

Figure 6B reveals comparison of the brain tissue IL-1β content in different tested groups. It was significantly increased in I/R+Veh group versus sham+Veh (*P* <0.001). Administration of lower doses of EA had no significant effects, while the 100 mg/kg EA (I/R+EA100) decreased it significantly compared to I/R+Veh group (*P*<0.001).

## Discussion

The results of current study showed that cerebral I/R by two vessel occlusion (2VO) causes elevation of inflammatory markers in brain tissue that followed by anxiety and depression -like locomotion, and exploratory behaviors impairment. Administration of higher dose of EA (100 mg/kg, gavages) after I/R induction for one week reversed tested behaviors and biomarkers toward the normal. 

Cerebral ischemia leads to neural disturbances such as motor, sensory, visual (33), speech and cognitive deficits, forgetfulness and impairment in cognition (34). Brain ischemia has known as one of the most debilitating brain events (35). It causes increased reactive oxygen species (ROS), and lipid per-oxidation thereby activating pathways leading to cell death in the vulnerable areas of the brain (36, 37). During a stroke, in addition to ischemia, overflow of the blood stream (reperfusion) also causes serious damages to the brain tissue (38). Our behavioral and biochemical findings are consistence with findings of other investigations. It shows that the current method to induction of 2VO cerebral I/R was performed accurately so that it could damage the brain function possibly by same mechanisms. 

Following cerebral ischemia, a cascade of molecular events caused BBB breakdown. It was evidenced that tight junctions between the brain endothelial cells are damaged after ischemic stroke (2). It has been shown that the BBB permeability after cerebral ischemia could initiate behavioral deficits. Therefore, lessening of the BBB permeability could attenuate behavioral impairments following cerebral ischemia (39). Results of the current study showed that EA decreased Evans blue outflow after cerebral ischemia, indicating that EA can attenuate BBB disrupted permeability. The protective effect of EA against BBB disruption may be mediated through inhibition of inflammatory cytokines. It was suggested that phenolic compounds could reduce deleterious effects of I/R injuries such as neurological deficits, cerebral water content and BBB permeability in animal model (39, 40).

The main mechanisms for damages during reperfusion are oxidative stress, leukocyte infiltration, platelet activation and accumulation, and excessive permeability of BBB, which ultimately leads to edema or stimulates hemorrhage (41). Thus, re-increasing of blood flow causes the hyper oxygenation thereby release of free radicals, and finally death of damaged cells. In addition, during reperfusion white blood cells are triggered to release inflammatory factors such as interleukins and free radicals in the damaged brain areas, and may occlude small blood vessels and cause more intense ischemia (42). According to some studies, with the occlusion of blood flow, brain energy sources are limited to the amount of stored compounds (43, 44). Our data also indicate that I/R induction caused the BBB disruption and thereby brain edema and inflammation of the brain followed by behavioral impairment. 

Since most of the energy consumed by the neurons is necessary to keep the ionic balance across the cell membrane (45), basic disturbances begin at the electrolyte balance in the cell (44). Eventually, neurons try to provide ATP through an anaerobic glycolysis, which leads to the production of lactic acid. It has also been shown that the loss of high-energy compounds such as ATP during ischemia, in addition to disrupting the amounts of sodium and potassium electrolytes, caused to speed the Ca^+2^ inward into the cell and extra elevation of intracellular calcium concentration and activation of phospholipase A2 and then protein kinas in cell membrane. It causes to catalyze hydrolysis of membrane lipids, leading to the production of free fatty acids and arachidonic acid, a precursor for prostaglandins production (45). In addition, other events occur such as increased activity of endonuclease, proteases, proteolytic enzymes, nitric oxide syntheses and oxygen species secondary to increasing intracellular calcium ions and consequently cell damage (46). In current work, behavioral impairments, and brain inflammatory deficit due to cerebral stroke improved in I/R rats treated with EA for 7 consecutive days. 

Inflammatory cytokines increased after cerebral ischemia causing brain damage and poor outcome in stroke patients (47). Cytokines rapidly increased in pathological conditions such as ischemic stroke (48), and increasing of the pro-inflammatory cytokines are correlated with a worse clinical outcome and a larger infarct size in animal models (49). It was reported that TNF-α and IL-6 levels were increased within hours following global ischemia (50). Results of the present study showed that cerebral ischemia and reperfusion could trigger inflammatory responses that manifested in increasing pro-inflammatory cytokines such as TNF-α and IL-1β. Our observations are consistent with other prior reports (51, 52). EA showed protective effects due to its anti-inflammatory properties (53, 54). A growing body of literature suggests the involvement of the TNF-α and IL-1β in the pathophysiology of cerebral ischemic injury (55, 56). Therefore, suppressing the TNF-α and IL-1β expression is effective for inhibiting cell death following cerebral ischemia (50). 

A study indicated that EA is able to prevent colon inflammation through intervention in the nuclear factor kappa beta (NF-kB) pathway, thereby reducing the inflammatory markers of cyclooxygenase 2 (COX-2), inducible nitric oxide synthase (iNOS), TNF-α and IL-1β. These effects can be linked to the antioxidant activity of the EA, which its chemical activity is through modulation (modification) of factors associated with inflammation. As a result, EA has anti-cancer and anti-inflammatory activities (57). TNF-α, interleukins 1 and 6, and COX2 are all regulated by NF-kB transcription factor that is affected by EA action (58). EA reduces the amount of brain infarction and apoptosis in brain through mechanisms such as protein apoptotic pathway adjustments, suppressing mitogen-activated protein kinase (MAPK) and provoking inflammatory mediators. MAPK cascades are associated with cell survival and apoptosis. In addition, the neural inflammation induced by hypoxic-ischemia potentially contributes to apoptosis adjusting the MAPK values and reducing the expression of the inflammatory proteins by EA that indicates its protective effects in hypoxic-ischemia (59). 

## Conclusion

Cerebral ischemia through the mechanisms of BBB disruption followed by increasing the brain water content (brain edema) and inflammatory cytokines in brain tissue caused significant behavioral disturbances. EA can be a promising therapeutic agent with low adverse effects for cerebral I/R disturbances. However, the discovery of the precise mechanisms of the effect of EA on related complications requires more extensive investigations. 
